# Towards safety4.0: A novel approach for flexible human-robot-interaction based on safety-related dynamic finite-state machine with multilayer operation modes

**DOI:** 10.3389/frobt.2022.1002226

**Published:** 2022-09-30

**Authors:** Mohamad Bdiwi, Ibrahim Al Naser, Jayanto Halim, Sophie Bauer, Paul Eichler, Steffen Ihlenfeldt

**Affiliations:** ^1^ Department of Cognitive Human-Machine-Systems, Fraunhofer Institute for Machine-Tools and Forming-Technology, Chemnitz, Germany; ^2^ Department of Production System and Factory Automation, Fraunhofer Institute for Machine-Tools and Forming-Technology, Chemnitz, Germany

**Keywords:** dynamic risk analysis, human-robot collaboration, human safety, industry 4.0, finite state machine

## Abstract

In the era of Industry 4.0 and agile manufacturing, the conventional methodologies for risk assessment, risk reduction, and safety procedures may not fulfill the End-User requirements, especially the SMEs with their product diversity and changeable production lines and processes. This work proposes a novel approach for planning and implementing safe and flexible Human-Robot-Interaction (HRI) workspaces using multilayer HRI operation modes. The collaborative operation modes are grouped in different clusters and categorized at various levels systematically. In addition to that, this work proposes a safety-related finite-state machine for describing the transitions between these modes dynamically and properly. The proposed approach is integrated into a new dynamic risk assessment tool as a promising solution toward a new safety horizon in line with industry 4.0.

## 1 Introduction

**FIGURE 1:**
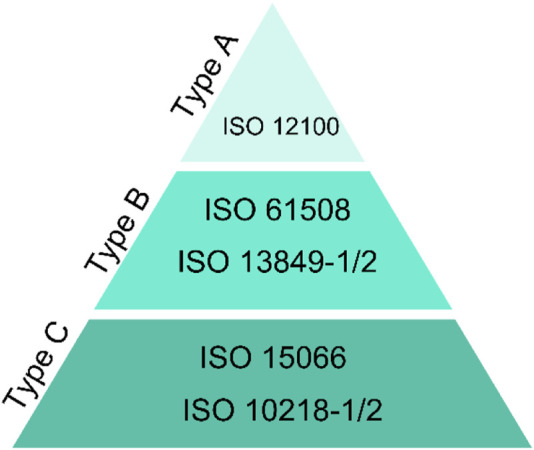
ISO types.

The CE conformity declaration (CE-marking according to the Machinery Directive 2006/42/EC) is the final mandatory step in Europe, which indicates that the machinery (e.g. the robot cell) meets European Union standards for health, safety, and environmental protection. Figure 1 shows the safety-related standards for the implementation of robotic systems according to 2006/42/EC. In general, the standards are divided into three categories: 1) Type A standards: describe the general principles of machinery design principles, 2) Type B standards: describe the generic safety standards covering safety aspects and safeguard across a wide range of machinery and 3) Type C standards: describe the safety standards for a specific machine group. ISO 12100:2010 (ISO 12100:2010, 2016) as basic safety standards (type A) define the basic terminologies and principles for achieving safety in the design of machinery. Furthermore, it specifies a methodology for risk assessment and risk reduction. The Type B standards are also generic safety standards. However, they cover specific safety aspects or one type of safeguard which can be used within a wide range of machinery. E.g. ISO 13849-1:2015 (ISO 13849-1:2015, 2015) provides the safety requirements and guidelines for designing and integrating safety-related parts of control systems. The ISO 13849-2:2012 (ISO 13849-2:2012, 2012) defines the procedures and conditions for validating the designed safety functions according to the ISO 13849-1. In the standards “Type C”, the safety requirements for a particular machine or group of machines are addressed in detail, e.g. ISO 10218-1:2021 (ISO 10218-1:2021, 2021) specifies requirements and guidelines for the inherent safe design, protective measures, and information for the use of industrial robots, while the ISO 10218-2:2021 (ISO 10218-2:2021, 2021) specifies safety requirements for the integration of industrial robots and industrial robot systems. ISO/TS 15066:2016 (ISO/TS 15066:2016, 2016) as technical specification defines safety requirements for collaborative industrial robot systems and the work environment. Revisions to the robotics-related standard are under process by the technical committee “ISO/TC 299”.

**FIGURE 2:**
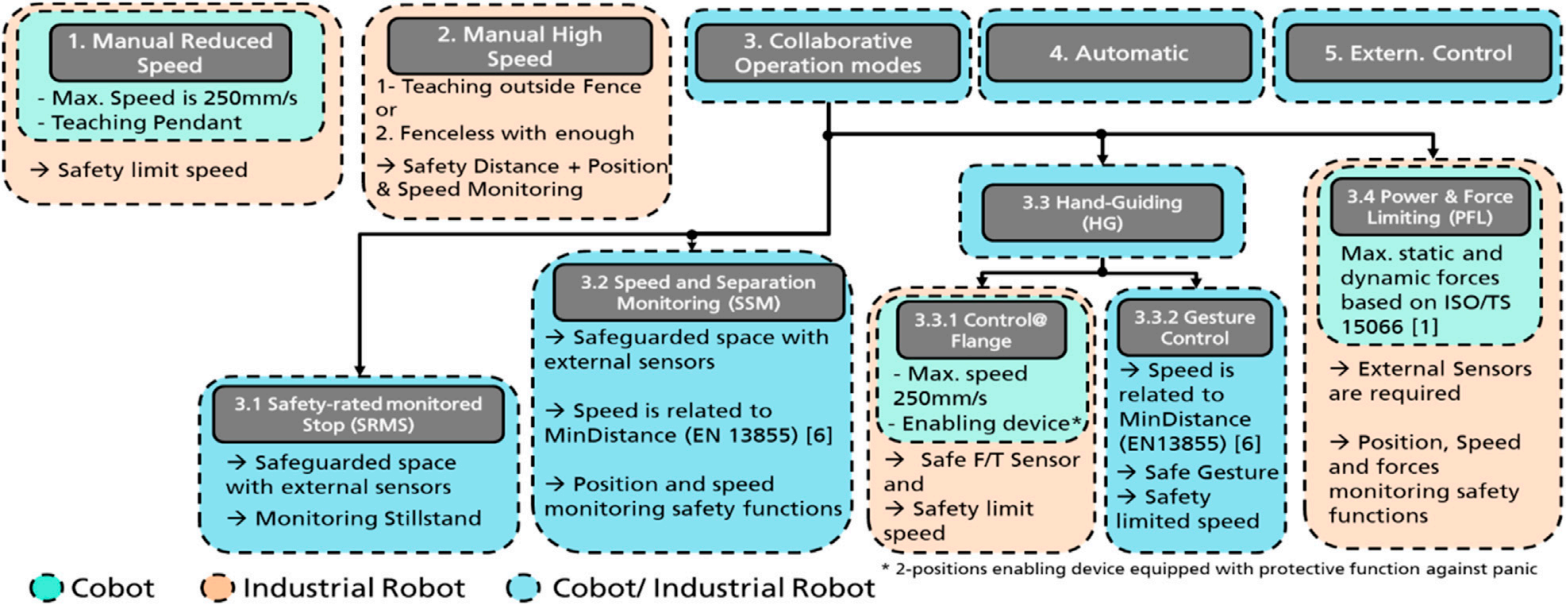
Possible Operation modes related to cobots and industrial robots.

According to the ISO/TS 15066:2016, “Safety-Rated Monitored Stop” (SRMS), “Speed and Separation Monitoring” (SSM), “Hand Guiding” (HG), and “Power and Force Limiting” (PFL) are the main four collaborative methods for collaborative operation (HRC). Figure 2 illustrates these operation modes and techniques for collaborative operation for industrial robot systems. In “SRMS”, the safety sensors directly stop the robot’s operation when a human enters the work cell respectively collaborative workspace. “SSM” mode allows the worker to be in the collaborative workspace while the robot moves by maintaining the protective distance between worker and robot. This method for collaborative operation ensures to stop the robot before any collision with the worker may occur. The safeguarded space should be monitored with external safety sensors (ISO 13855:2010, 2010) provides the basis for positioning the safeguards taking into account the speed of parts of the human body. Various approaches for “SSM” considering dynamic speed regulation of robots are presented in (Byner et al., 2019), (Kolbeinsson et al., 2018), and (Michalos et al., 2015). In another work (Rashid et al., 2020), the minimum protective distance between worker and robot has been addressed in details. Furthermore, the system performance in scenario with heavy-duty robot has been systematically investigated.

As is shown in Figure 2, during “HG” collaborative mode: the worker can use a hand-operated device “Control@Flange” or even gesture “GestureControl” to control the motion of the robot. HG requires additional PFL or SRMS safety functions. Most of the cobots possess integrated hand-guiding functionalities. However, the industrial robot systems require an external safe force/torque sensor or hand-guided system to measure the worker’s applied forces and torques. In “PFL”, the robot system may come into direct contact either intentionally or accidentally with the worker. However, the contact power and force should be limited to a safe level to reduce risk and avoid harming humans. Most of the implemented applications with PFL operation mode are currently realized based on lightweight “cobots”, being industrial robots constructed for it and equipped with PFL safety functions. PFL in high payload HRC applications is still difficult to implement due to the high inertial mass of the robotic itself and potentially dangerous collisions. In general, the safety requirements for these methods are still under development for the upcoming revision of ISO 10218 (to be published in 2022).

Some industrial robot systems offer two manual operation modes for teaching or commissioning processes. The teaching process in the first operation mode can be performed without any barriers but with reduced speed (max. 250 mm/s). While in the second operation mode, the worker should stay outside the cell to let the robot moves at full speed (e.g. 2 m/s). During the teaching phase, the cobots usually work with the reduced manual operation mode. The operation modes “4” and “5” are typically used in the fully-automated tasks. In the fifth mode, an external control system, “e.g. Programmable-Logic-Controller (PLC)”, is used as a master control unit.

The operation mode is usually the primary key element for further steps during risk analysis, risk reduction, and defining the required safety sensors/functions. The Safety-related sensors/functions significantly impact the design of HRC applications in terms of efficiency and flexibility. Any change in the process, workflow, product, layout, etc., requires a new identification of possible hazards. Furthermore, the whole procedure for getting the CE marking could be repeated from scratch. Such kinds of policies are very cumbersome for both systems integrator and operator. They are almost no longer possible in the era of Industry 4.0, where agile manufacturing systems, flexible layout, dynamic processes, and customized product features are the main characteristics. In addition, myriad applications could require multiple sequential operation modes on the same cell to efficiently fulfill the required task, which is currently not feasible due to the restricted safety producers and lack of acceptable safety-related solutions. This work proposes a new approach for multilayer HRI operation modes merged with various process-related human-robot-interaction levels systematically. The proposed system is implemented using a dynamic finite state machine architecture. The following section will present an overview of the state of the art. Section 3 will illustrate the proposed approach in detail, while Section 4 explains one use case as an example for presenting the advantages of the proposed approach in reality. Section 5 will focus on integrating the proposed methodology in a newly developed risk assessment tool as a final result of this work. Finally, the proposed work will be concluded in the last section.

## 2 State of the art

As mentioned, the collaborative operation modes describe the interaction between humans and robots from a safety point of view. This focus makes executing the collaborative operation modes in the applications very tricky. In other words, there is a large gap between process-related and safety-related functionalities during the design phase of the collaborative tasks. Furthermore, the complexity of safety design forces the safety planner to consider the operation modes individually for fixed tasks. Any changes in the operation modes or even the process could require new certification procedures. In addition, when various interactions between humans and robots are desirable, implementing multiple operation modes is laborious. This research problem has initiated researchers worldwide to exploit the approaches to ensure HRI at the implementation level (from the process point of view). A general insight of the current framework and state of the art in the implementation level for safety in industrial robotic environments has been reviewed by S. Robla-Gomez et al. (Robla-Gomez et al., 2017). Even though this review shows many possibilities to implement safeguarding with sensors, it does not mention any standards or metrics at the implementation level that could bridge the safety operation modes with the functional safety of the machinery. Some quantitative metrics have been introduced in several works (Galin et al., 2020), (Kolbeinsson et al., 2018), (Marvel et al., 2020). Kolbeinsson et al. have suggested a metric by visualizing interaction level in HRI based on human and robot efforts (Kolbeinsson et al., 2018). Marvel et al. and Aaltonen et al., propose other metrics as quantiative measurmements in the design of the HRI process (Marvel et al., 2020) and (Aaltonen et al., 2018). Galin and Mescheryakov proposed the quantified process parameters for HRI regarding efficiency (Galin et al., 2020). Although these approaches have covered the quantification and validation process to benchmark the HRI design and process, the metrics are still far from implementation. An approach to validate safety in HRI has been introduced by Valori et al. by introducing safety skills and their validation protocols (Valori et al., 2021). Although the safety skills have been tested under strict validation protocols to reduce specific risks, the safety skills are limited to simple tasks. The protocols are more into validation measurements for testing purposes than implementation purposes. Michalos et al. have introduced and suggested an approach to ensure safety in HRI by considering and combining highlighted functional safety, safety operation modes, and machinery directive based on the shared tasks and workspace at the implementation level (Michalos et al., 2015). Unfortunately, there is no general overview of which complementary functional safety, safety operation mode, and machinery directive are required for different tasks or interactions between humans and robots. This open point can also be found in Askarpour et al.'s method, which uses a complex non-deterministic formal model resulting from human errors (Askarpour et al., 2017). In the latest work of Gualtieri et al. (2022), guidelines to develop and validate safety aspects in the HRI workspace have been suggested. This method covers a quantification methodology for the possible mechanical hazards in the design of the HRI workspace and suggests risk assessment strategies based on the standards. Although the methodology has a good scope to cover the development of safety measures in HRI for non-expert users, the method only focuses on the assembly process.

**FIGURE 3:**
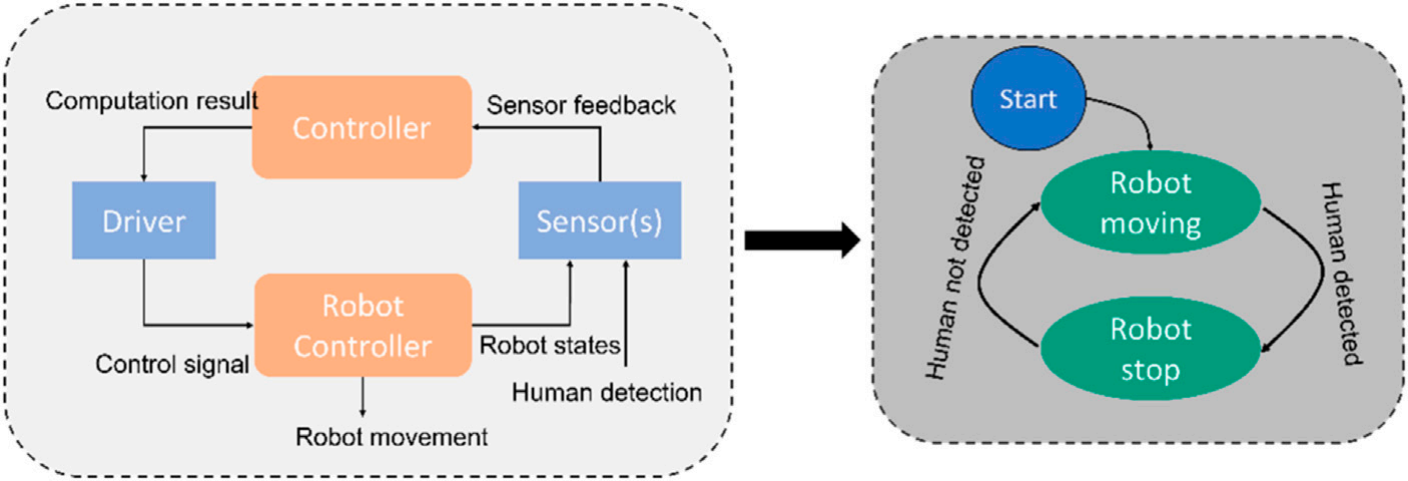
Comparison between an STPA diagram (left) and FSM diagram (right) for a fictional SRMS use case.

The approach’s overview and classification methodology of this work has been exploited in a previous paper (Bdiwi et al., 2017) by introducing a new classification methodology for HRI applications using four interaction levels. *Via* the proposed four levels of interaction, most of the possible HRI applications in the industry could be classified. Furthermore, the safety procedures and safe zones could be derived based on single or even clustered safety operation modes for each interaction level. Even though work has focused on improving the safety procedures for HRI; a general system architecture was missing for quantifying the complex safety functionalities in the level of interactions. The finite state machine (FSM) is one effective method to implement a technical system with a minor development curve. FSM offers a very effective method in the implementation of complex robot behaviors in comparison to monolithic programming. The system’s sequential, deterministic, and causal behaviours ease the implemented robotic system for debugging, modification, and enhancement. Balogh and Obdrzalek have introduced these benefits (Balogh et al., 2018). Although FSM would offer many simplifications in developing and implementing the robotics system, design errors, cognitively complex human decision-making errors, and other failures are challenging to observe. Another possible approach to identify these new causal factors is using a top-down analysis called system theoretic process analysis (STPA), a method used in a technical system to show its behavior and address component interactionfailures. Integrating between STPA and FSM could assist the safety analysis in identifying the dysfunctional behavior of a system (Abdulkhaleq et al., 2013). However, the proposed approach aims to couple the process-related classification (Interaction-levels) with the safety-related classification (operation modes) to reduce the effort and complexity during risk analysis and mitigation. A general comparison between STPA and FSM is shown in Figure 3. Figure 3 left shows the intermodule communication in a SRMS collaborative operation. In this figure, all of the data flows can be seen in the STPA diagram. When failures occur, the failure analysis can be performed by checking the causality in the control structure. Figure 3 right depicts the FSM diagram for the same use-case. In contrast to STPA, FSM method is focused on the causality of the system state. Each state (action) will be triggered by a deterministic signal. Hence, it can be concluded that STPA has a general focus on analyzing failure in the hardware and communication level. In contrast to STPA, FSM focuses the debugging level in the functionality of the system. Hence, this work focuses on the FSM more than STPA for hardware integration. This approach allows the user also to plan and implement complex and agile collaborative tasks flexibly and intuitively.

## 3 The proposed approach

**FIGURE 4:**
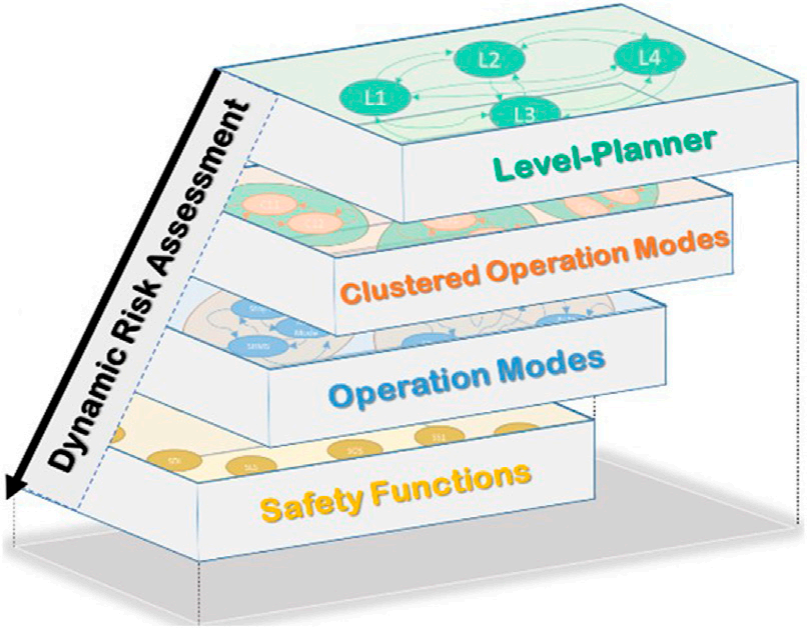
Structure of the proposed approach.

Figure 4 illustrates the main structure of the proposed approach. The first layer “Level-Planner”, facilitates the classification of the proposed application according to the interaction level (Bdiwi et al., 2017). The second layer presents a combination of possible clustered operation modes to fulfill the described task in the level-planner. Every cluster consists of various operation modes shown in the third layer and modeled using a finite state machine. The fourth layer contains all the required safety functions for every operation mode. All layers are systematically coupled to ensure the system’s legibility and the user’s comprehend-ability. They will be described in detail in the following sections.

### 3.1 Level-planer

As (Bdiwi et al., 2017) explained, the main difference between interaction level 1 and interaction level 2 is that the human presence is not intended during the normal process of level 1. In other words, there is no shared task in level 1, and the human enters the robot cell only, e.g. in emergencies. In level 2, the robot and humans work on a shared task. However, the cooperation is still low. At this level, the robot can move toward a predefined position during the human presence, taking into account the safety distance and type of collaborative operation modes. Level 3 requires active robot control during the shared task, where the robot can change its position and react to human movements. The possible shared tasks in this level are, e.g. Handing-over, Gesture-based control, or even automatic path planning. In path planning, the robot can automatically change the working height based on human anthropometry or regenerate a new path during the shared task to avoid any collision with the human. In Level 4, physical HRI is necessary to fulfill the task. For instance, the robot can bring heavy components to the human or a predefined position near the assembly line; then, the human can guide the robot to a final position by using human forces (Handguiding-System). In this work, the proposed interaction levels are focused on the implementation of HRC with serial manipulators. It is also possible to implement the proposed method for mobile manipulation by extending the safety functions with safety related standards and control system e.g. (Elhaki and Shojaei, 2020) and (Elhaki and Shojaei, 2022) for mobile robotic.

**FIGURE 5:**
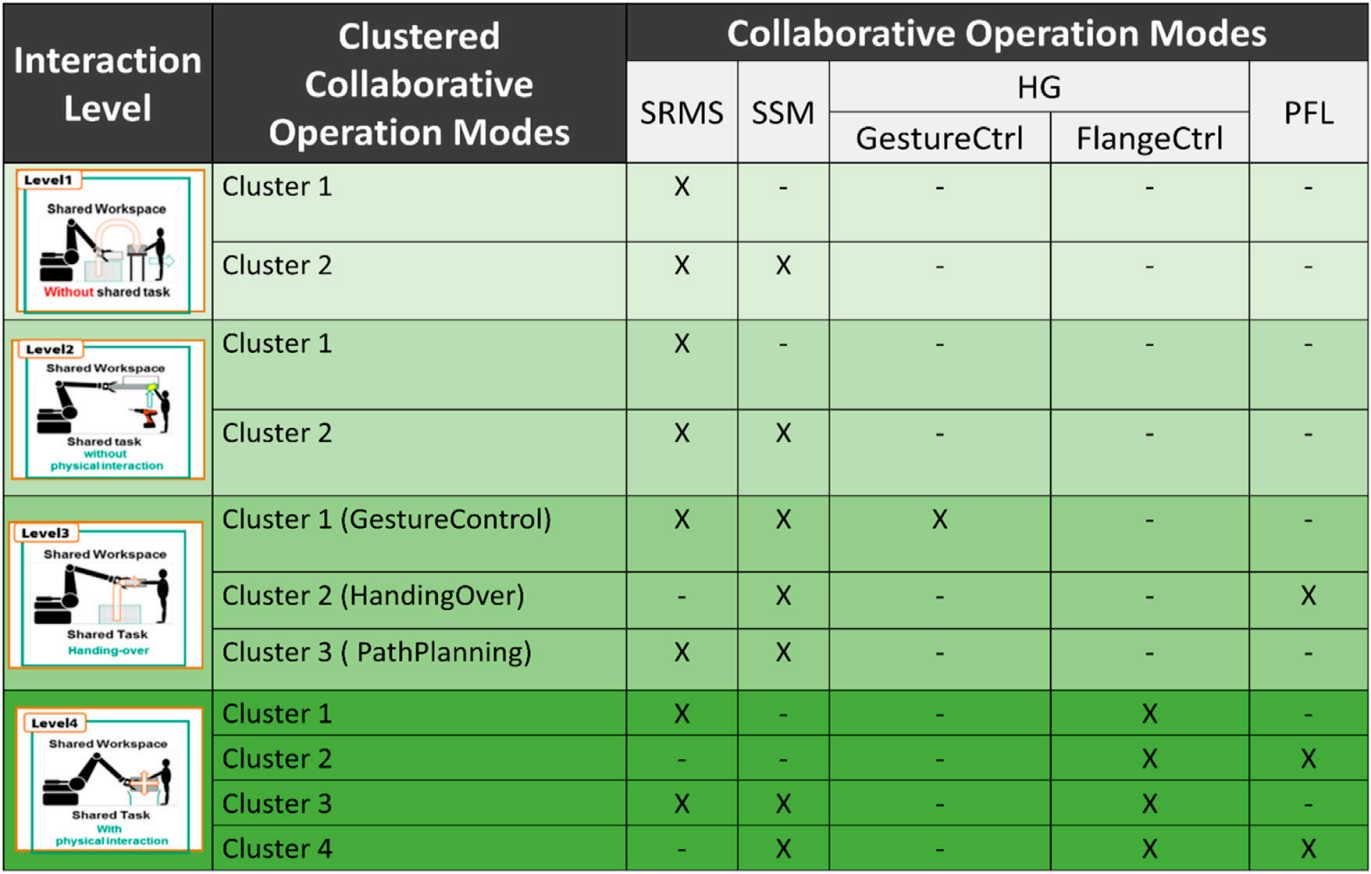
Some possible examples for clustered collaborative operation modes in every.

Figure 5 illustrates the possible collaborative operation modes at each interaction level. **Level 1** can contain, e.g. two clusters. **Cluster 1**: SRMS-Standalone: the whole workspace and work process will be performed in SRMS mode. **Cluster 2**: SRMS + SSM: this cluster allows the robot to change its speed according to the human position in some process or even defined zones of the workspaces. As is already mentioned, the human enters the robot cell only, e.g. in emergencies. Hence, when the human enters any dangerous area monitored by the safety sensors, the robot will have to stop in stop-category 1. Therefore, user confirmation is required for activating the robot again. Cluster 2 allows the user to plan different collaborative operation modes in different zones (e.g. SSM for the entrance areas besides the footpaths to avoid any unnecessary robot stops, while the SRMS can cover more critical areas). Each cluster has its safety functions which will be described more in the next section. **Level 2** could also consist of the same clusters. However, the safety functions are different, as is shown in Supplementary Appendix S2. At this level, the human is intended to participate in the process during the shared task. The robot should be able to restart automatically without an additional confirmation by the worker. Hence, the robot will have to stop in stop-category 2 when the human enters the danger areas. Thestop-category 2 will increase the efficiency and availability of the facility. Figure 5 also presents three possible clusters of collaborative operation modes in **level 3**. **Cluster 1** “GestureCtrl” gives the users the possibility to control the robot based on their gestures. The user can perform these procedures within the Handguiding “HG” (GestureControl in Figure 2 and as a shortcut GestureCtrl in Figure 4) operation mode. Usually, gesture control is performed during a specific task during the whole process or even in a defined and restricted area. The cluster that is mixing SSM, and SRMS with HG-GestureCtrl could be very useful for increasing the facility’s efficiency and flexibility on the one hand. On the other hand, it ensures the safety of the human during all processes with the required safety functions, no more, no less. **The second cluster** “Handing-Over” could be used in any process which contains handing-over tasks between humans and robots. This cluster can combine, e.g. SSM with PFL, to ensure that the maximum possible impact forces/torques during the collision will not increase the maximum described values in ISO/TS 15066. **The third cluster**, “PathPlanning”, requires additional safety functions for monitoring the paths during the shared task. In **level 4**, four possible clusters are presented. In the cobots applications, the HG-FlangCntrl can be combined with PFL **(e.g. cluster2)** or with modes, SSM, and PFL **(e.g. cluster4)**. The HG-FlangCntrl can be combined with SRMS **(e.g. cluster 1)** or with SRMS and SSR **(e.g. cluster3)** in the heavy-duty application. PFL in high payload HRC applications is still difficult to implement due to the high inertial mass of the robotic system and potentially dangerous collisions. The clusters mentioned previously are built based on interaction levels (Bdiwi et al., 2017). These clusters were chosen as examples of common scenarios. However, unusual scenarios or special requirements may be realized based on the level of interaction with the new cluster. The new cluster defines the state graph and which safety functions could be involved.

### 3.2 Multilayer collaborative operation modes

**FIGURE 6:**
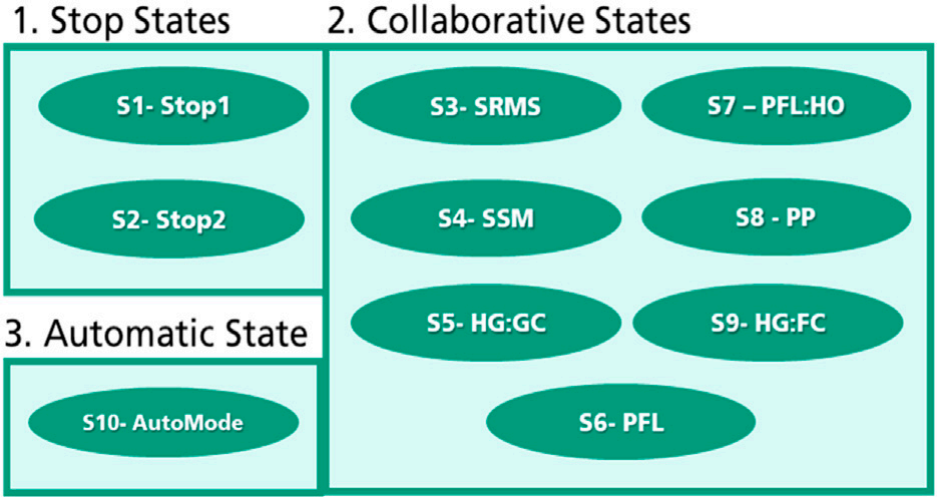
Machine states.

In the proposed approach, every collaborative operation mode will represent one machine state to build a safety-related finite state machine properly. These collaborative states are; S3-SRMS, S4-SSM, S5-HG: GC, S6-PFL, S7-PFL: HO, S8-PP, and S9-HG: FC. S3 (SRMS) and S4 (SSM) are already well explained in the first section. S5 (HG: GC) represents the state of Handguiding using gesture control in level 3, while the S9 (HG: FC) represents the hand guiding use, e.g. hand guiding device mounted on the robot flanch. S7 (PFL: HO) is a special collaborative state for handing-over tasks based on PFL operation modes in the third interaction level. S8 (PP) is also a particular collaborative state during the third interaction level when the robot can modify its paths. All the possible proposed machine states are shown in Figure 6. In addition to that, two states represent the stop categories “S1 (Stop1) and S2 (Stop 2)”, and one state represents the automatic mode “S10 (AutoMode)”. All the previously mentioned collaborative operation modes can be integrated to create variable clusters concerning the interaction levels.

**FIGURE 7:**
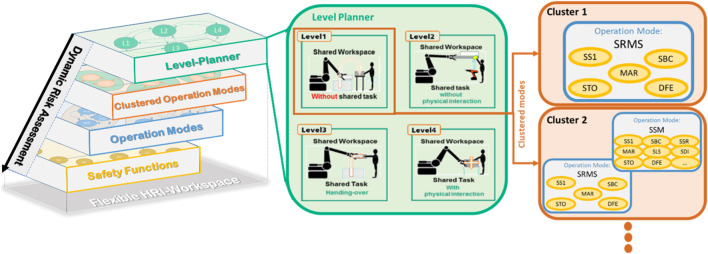
Example of the possible collaborative operation mode clusters of level 1 with their safety functions.

Figure 7 illustrates an example of the possible clusters in level 1. Furthermore, it presents the required safety functions of each cluster. All the safety functions and their acronyms are described in Supplementary Appendix S1. The transition and state conditions between the operation modes can be coupled with the safety functions using the finite state machine structure. More details will come in the next two sections.

### 3.3 Enhanced safety functions

**FIGURE 8:**
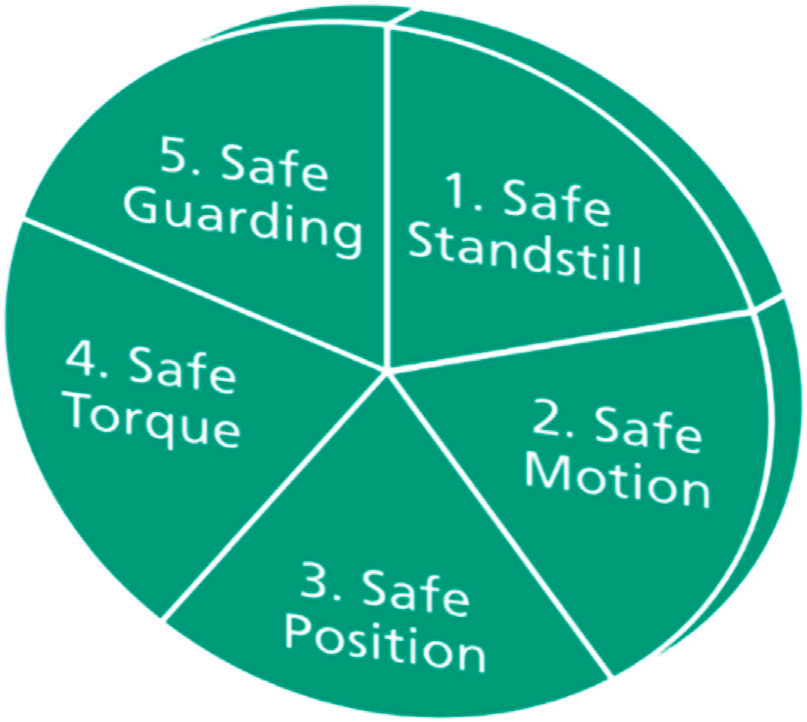
Overview of main categories of the safety functions.

As is already in section 3.1 presented, each level has various clusters of operation modes consisting of a bundle of safety functions. The basic safety functions are listed in DIN EN IEC 61800-5-2:2017 (DIN EN IEC 61800-5-2:2017-11, 2017). Figure 8 shows the main categories of the required safety functions in robotics applications. The functionalities of all safety functions in these categories are described in Supplementary Appendix S1. The first category is Safe Standstill. It contains all the safety functions for controlling the monitoring the robot during the standstill. These functions are; 1. Safety Stop1 (SS1), 2. Safe Brake Control (SBC), 3. Safe Torque off (STO), 4. Safe Stop2 (SS2) and 5. Safe Operation Stop (SOS). The safety functions in the second category “Safe Motion” are responsible for controlling and monitoring the robot’s motion, e.g. 1. Safety Limited Speed (SLS) 2. Safe Speed Monitoring (SSM), 3. Safety Range Speed (SRS), 4. Safe Direction (SDI). The third category of safety function takes care of the robot positions, e.g. 1. Safety Limited Position (SLP) and 2. Safe Cam (SCA). The safety function in the fourth category is the Safe Limited Torque (SLT) for preventing the actuator from exceeding the torque limit. This work proposed also enhanced safety functions within the fifth category, “Safe Guarding”, for controlling and monitoring the interaction between the human and the robot. e.g. 1. Dangerfield-Entry (DFE), 2. MinDistance (MiD), 3. Coopfield_Entry (CFE1), 4. ManualRes (MAR) 5. OperationRes (OPR), 6. GestureCntrlStart (GCS), 7. GestureCntrlEnd (GCE) etc. The whole list of the enhanced safety functions is listed in Supplementary Appendix S1. The structure of the proposed approach is dynamic. Hence, it allows the user to extend the bundle of the safety functions if it is required.

### 3.4 Safety-related finite-state machine for collaborative applications

**FIGURE 9:**
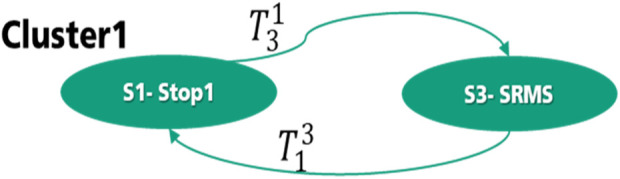
States graph of cluster 1—level 1.

After presenting the main safety functions and the possible states of the collaborative operation modes, this section will show the main concept of the safety-related finite-state machine for collaborative applications. A transition $T_{m}^{n}$ presents the transition from the start state $S_{n}$ to the end state $S_{m}$ . Every transition $T_{m}^{n}$ consists of a couple of conditions representing the relation between safety functions and the machine state to switch from the start state to the end state. The transition, which contains a set of safety functions with the AND ( ∧ ) operator, is true if all its safety functions are true. While the transition with Or ( ∨ ) operator is true if only one of its safety functions is true. For better illustration, the finite state machine of both collaborative clusters in the first level of interaction will be explained in this section. As is shown in Figure 9, there are two states, S1 (Stop1) and S3 (SRMS), in cluster 1 of level 1. There are two transitions, $T_{3}^{1}$ and $T_{1}^{3}$ , between S1 and S3. By supposing that S3 is the start state, the robot goes in S1, when the human enters the danger area for an emergency situation. This happens when the safety function “Dangerfield_Entry DFE ” is active. Hence, the safety functions SS1 for maintaining the position of the actuator, STO for disabling the torque in the actuator, and SBC for supplying a safe output signal to drive an external brake system should also be active to transfer the machine from the S3-state to the S1-state. These transition conditions are presented in Eq. 1.

$$T_{1}^{3}\to \left(DFE\wedge SS1\wedge SBC\wedge STO\right)$$ (1)

The transition condition from S1-state to S3-State consists of two functions: 1. $\bar{DFE}$ this means that the Dangerfield_Entry DFE is deactivated, and the human has left the danger area. Besides that, a user confirmation through MAR “Manual restart button” is necessary for the Stop1 to ensure that the process could be continued after the inexistence of the human in the danger area, as shown in Eq. 2.

$$T_{3}^{1}\to \left(\bar{DFE}\wedge MAR\right)$$ (2)

**FIGURE 10:**
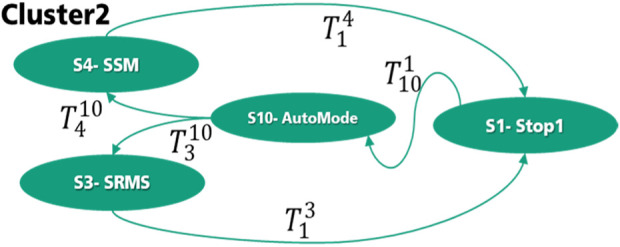
State graph of Cluster 2—Level 1.

As is shown in Figure 10, the second cluster of Level 1 has a more complex state graph which consists of 4 states. In this cluster, two other states have been added. S10 (AutoMode) represents the automation mode which can be the start mode. $T_{3}^{10}$ and $T_{4}^{10}$ represent two transitions from the automatic mode to both collaborative mode SSM and SRMS. SSM can start when the human enters the collaboration areas by activating the safety functions (Collaborative_Field_Entry CFE). Various speed limitations can be defined in different collaborative fields (e.g. from CFE1 until CFEX). The safety functions, which are listed in Supplementary Appendix S1 in the safe motion, should also be active, as is shown in Eq. 3.

$$T_{4}^{10}\to \left(\left(CFE1\vee CFE2\ldots CFEX\right)\wedge SLS\wedge SSM\wedge SSR\wedge SDI\right)$$ (3)

The transitions $T_{1}^{4}$ and $T_{1}^{3}$ from S4 and S3 states to the S1-state happen when the DFE is activated, and all the required safety functions of Stop1 are also activated, as is shown in Eqs 4, 5. By transition $T_{1}^{4}$ , if one of the safe motion functions is not active (e.g. the actuator speed exceeds the maximum speed limit), the robot goes to S1-state, as is shown in Eq. 5.

$$T_{1}^{3}\to \left(DFE\right)\wedge SS1\wedge SBC\wedge STO$$ (4)

$$T_{1}^{4}\to \left(DFE\;\right)\vee \left(\bar{SLS}\vee \bar{SSM\;}\vee \bar{SSR}\vee \bar{SDI}\right)\wedge SS1\wedge SBC\wedge STO$$ (5)

The transition $T_{10}^{1}$ from the S1-Stop1 to the S10 automatic mode happens when nobody is inside the danger area. The user confirms it through the safety function operation restart (OPR), as shown in Eq. 6.

$$T_{10}^{1}\to \left(\bar{DFE}\wedge OPR\right)$$ (6)

When the transition to the automatic mode happens successfully, the user can confirm further through MAR “Manual restart button” to start the SRMS operation mode, as shown in Eq. 7.

$$T_{3}^{10}\to \left(\bar{DFE}\wedge MAR\right)$$ (7)

The manual confirmations at this level are necessary because the human can enter only in emergencies, and it is not a part of the process. In the second level of interaction, these manual confirmations are not required. However, the same principle of the safety-related finite-state machine can be generalized to all other clusters at all levels of interaction. The state graphs and their related transitions of all other clusters are presented in Supplementary Appendix S3. The state graphs in this novel approach represent the clustered collaborative operation modes from the proposed method. The states are selected by considering the defined collaborative operations (ISO 15066) and related safety functions (IEC/ISO 61508). When additional or customized safety functions are integrated in the cluster, the states of the state machines must be extended to represent the additional extension.

## 4 Case-study

**FIGURE 11:**
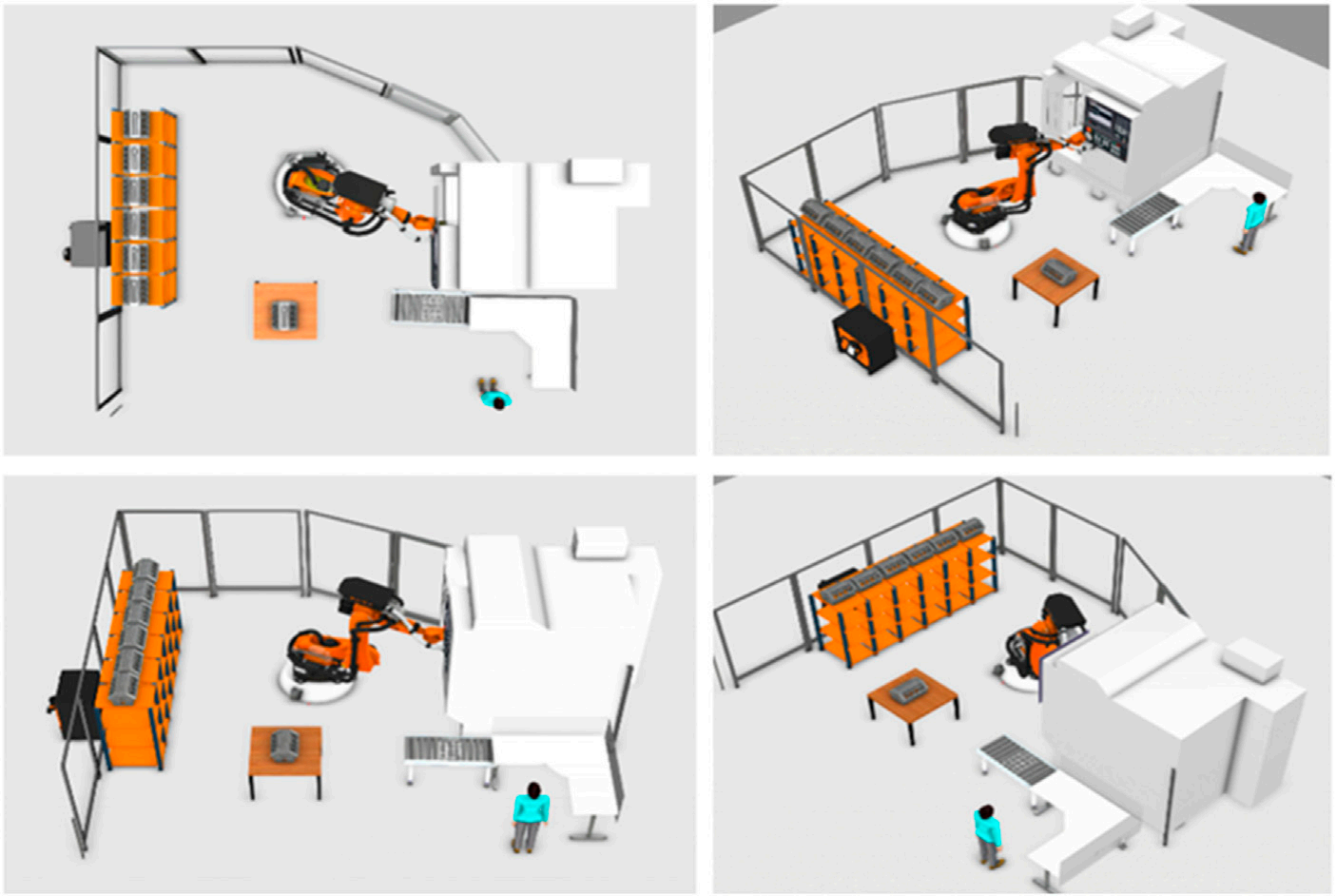
Fenceless manufacturing (Human, Machine and Robot).

This section will present the application of the collaborative mode clusters through a practical example. Furthermore, it illustrates how the proposed approach can increase the efficiency of the process and ensure the safety of humans in every process. In this use case, handling and machining of a car engine (**collaborative machine tending)** are performed using a CNC machine EMAG VMC 300 MT integrated with a heavy-duty robot Kuka KR-180 Prime 2,900. The robot transfers the engine to different places as it is a heavy task for a human. Figure 11 illustrates the cell layout with the locations of the robot and machine. Additionally, a convey and two tables exist for the quality checking process. A storage place is needed on the left side of the cell where the finished items are placed there.

The scenario is as follows:

1- The storage place has unfinished pieces that the robot is programmed to take from the storage place in a known sequence.
2- A robot is responsible for moving the item from the storage place to the CNC machine. The robot places the item slowly, and the machine can fix the item to process.
3- The machine processes the item for creating screw holes and, it takes 10 min for each item.
4- After the process, the machine opens the safety door while robots move toward the machine. The robot grabs the item from the machine.
5- The quality process is a cooperation between humans and robots. The robot brings the item to the middle table, and the human starts checking the quality of the CNC process. Based on a command from the human, the robot moves instead to the storage place or towards the convey for rechecking and maintenance station.
6- The robot moves from the conveyor point to bring a new item from the storage place, or the robot will be there already if the quality checking is fine. Finally, the process repeats itself till the items are finished.

Such a scenario incorporates the human and the robot together to perform the process effectively. Furthermore, the human risk in such a cell will be high compared to a normal cell where human skill is not required. Hence, a proper risk assessment procedure could reduce the risk to humans in this collaborative environment.

**FIGURE 12:**
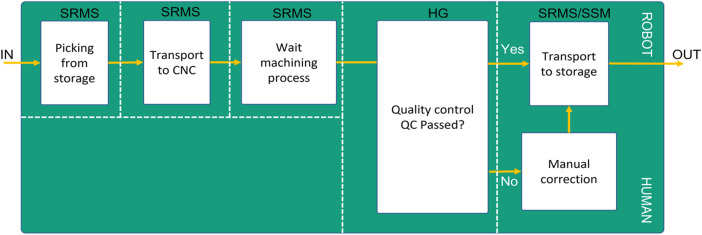
Use-Case Process-Flow concerning the operation mode.

In this use case, the interaction between humans and robots can be clustered under Level 3 or Level 4. It depends on the type of hand-guiding concept during the quality process. Here, the human can control and rotate the robot through camera-based neutral gestures (level 3) or through a hand-guiding device on the robot flange (level 4) to check the engine’s quality from different perspectives. As shown in Figure 12, the robot can work under SRMS operation mode while picking the item from storage, transporting it to the CNC, and waiting for the machining process. When the quality process starts, the robot can switch to the HandGuiding operation mode. During the final process, the robot can work under SSM while transporting the item to the maintenance station or under SRMS when the robot should transport the item to the storage back if the item’s quality is fine. Choosing the robot type and operation mode in such applications depends on many factors (Schneider et al., 2020), e.g. batch size, processing time by the machine, transporting time by the robot, required time for the manual tasks performed by humans. Using the proposed approach, can the user design the safety procedures of such agile and dynamic environments flexibly and adequately.

## 5 Dynamic risk assessment

**FIGURE 13:**
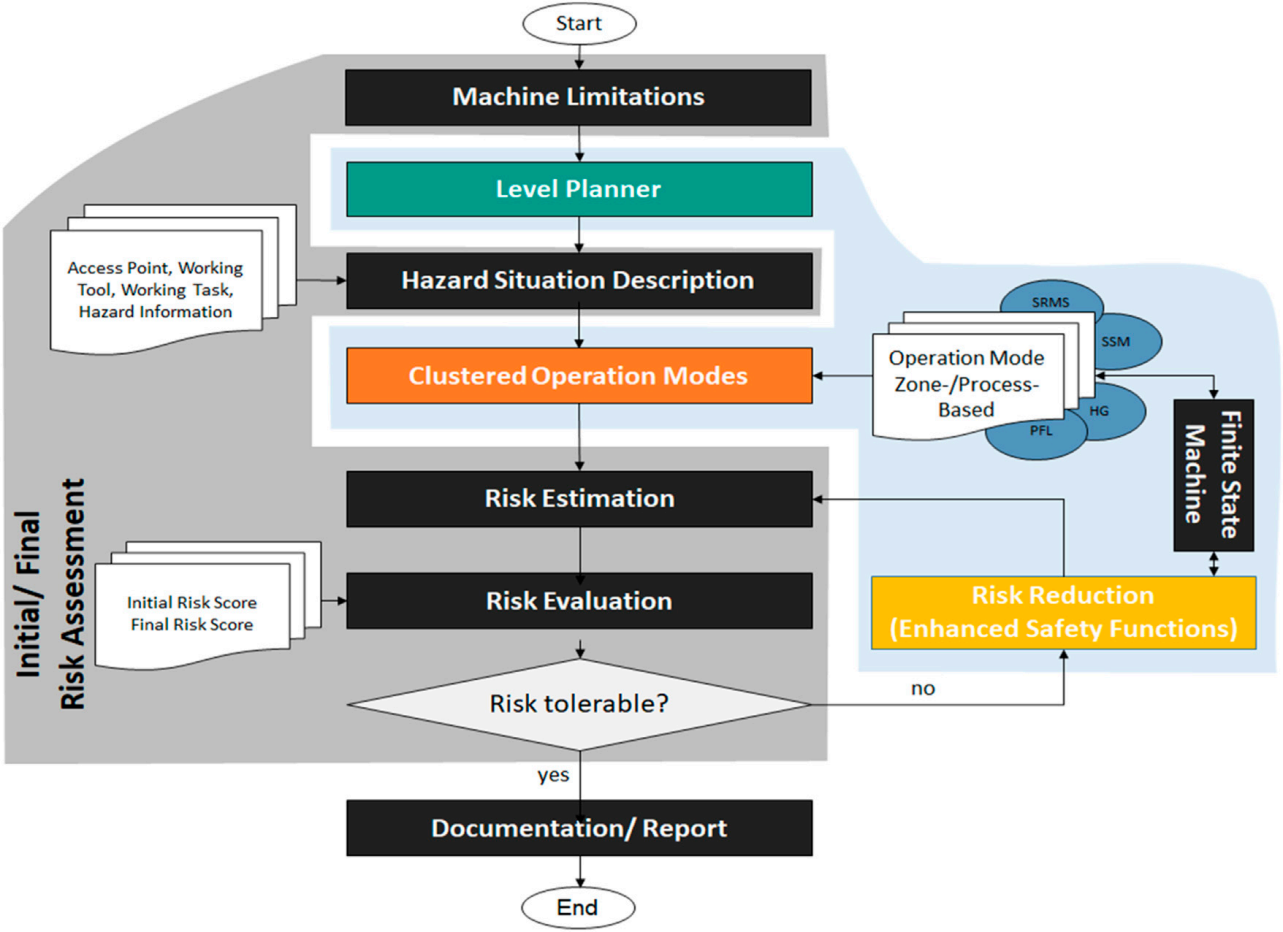
Integration of finite state machine with dynamic risk assessment methodology (Steps of the proposed risk assessment based on ISO 13849-1 and ISO 12100).

In order to reduce the risks in HRI robot cells, three management strategies include 1) Inherently safe design, 2) Guards and protective devices, and 3) administrative information, as are written in the ISO 12100. The most related paper (Realyvásquez-Vargas et al., 2019) in risks assessment for HRI addressed the first both strategies of risk management. These strategies are only implementable on their predefined use-case. Otherwise, other works give only a theoretical overview and metrics for risk assessment (Franklin et al., 2020) and (Hanna et al., 2020). Compared to these works, the proposed approach is generic and it can be implemented in any safety control device. Furthermore, it simplifies for the end-user the derivation of technical risk management in HRI-context by classifying HRI based on shared workspaces and tasks. Each cluster represents a use-case scenario regarding the requirement of the user. Hence, the functional safety inside the cluster can be customised depending on the user’s requirements. The recent study of Hornung und Wurll (Hornung and Wurll, 2021) addresses that lack of know-how and skills are the biggest hindrances in implementing collaborative robot systems. Figure 13 illustrates the integration of the proposed approach within the risk assessment methodology derived from ISO 12100 and ISO 13849-1. It extends the typical risk assessment as presented in grey color. The proposed approach begins with the determination of the machine limits, taking into account all the phases of the machinery life to fit the requirements of agile and flexible human-robot applications. The first step describes the machine’s characteristics, performances, and limits in an integrated process. Using the proposed level-planner, the user can classify all the planned processes according to the level of interaction with that machine. This procedure allows the user to easily estimate all possible hazards and all access points during that level of interaction. With the help of the clustered operation modes (zone-based, time-based), the user can define the machine movement range, space for people interacting with machines (operators, maintainers), the required time for each process, and the relation between this information and the operation modes properly. To assess the initial risk for each access point in the form of a risk score and to calculate the required performance level of each safety function, one needs to know the number of persons involved affected by the hazard, the duration, and frequency of the hazard exposure, the probability of occurrence, and the injury severity of the possible human injury.

After evaluating all initial risks, the risk reduction procedures start with its typical three steps, as mentioned previously. The innovative part of this approach focuses on the second step of risk reduction. In other words, it integrates the developed safety-related finite state machine with the safety functions of the guards and protective devices and with the collaborative operations. In general, the limitation of safety sensors should be considered during the risk management. Finally, if the risk is tolerable, the user can automatically generate all the required technical documentation for the declaration of conformity. The user can anytime reconfigure the safety design and risk assessment according to changes in the process, layout, or product.

## 6 Conclusion

As is already presented, the typical risk assessment procedures are very complex, and take a lot of time and effort. Hence, an expert must follow the implementation of the risk-assessment rules as written in the safety standards and guidelines. The expert should have a broad knowledge of the available safety functions and safe workspace methods. The complex, rigid and static procedures could lead to mistakes even by experts, and the state-of-the-art methodologies prevent them from planning and realizing agile production systems. The proposed approach establishes a dynamic and safe relation between the interaction levels, operation modes, and risk reduction procedures (safety functions). A safety-related finite-state machine has been illustrated for the transitions between these modes dynamically and adequately. Collaborative machine-tending has been described as a use case. Finally, the proposed approach has been integrated into a new dynamic risk assessment methodology as a promising solution toward a new safety horizon in line with industry 4.0.

## Data Availability

The raw data supporting the conclusion of this article will be made available by the authors, without undue reservation.
