# Supplementary material for: Towards safety4.0: A novel approach for flexible human-robot-interaction based on safety-related dynamic finite-state machine with multilayer operation modes
Source: Front Robot AI. 2022 Sep 30;9:1002226. doi: 10.3389/frobt.2022.1002226 (PMC9574623; doi:10.3389/frobt.2022.1002226)
Supplement: Supplementary file 1 [file DataSheet1.pdf]

Appendix I – Safety functions

Sector	Name	Description
Safe Standstill	Safety Stop1 (SS1)	Maintaining the position of the actuator to guarantee a controlled stop. The torque in the actuator is disabled (STO triggered).
	Safety Stop 2 (SS2)	Maintaining the position of the actuator at standstill. The standstill position is held precisely and can be continued after lifting the
	Safe Operating Stop (SOS)	Preventing leaving of the position while the drive control function is turned off / maintained.
	Safe Torque Off (STO)	Immediate removal of power to the machine actuators. Torque will be disabled by switching off the energy in the power supply
	Safe Brake Control (SBC)	Supplying a safe output signal to drive an external brake system.
Safe Motion	Safe Direction (SDI)	Monitor the actuator not to move in an invalid direction.
	Safely Limited Speed (SLS)	Preventing the actuator to exceed the maximum speed limit.
	Safety Speed Monitoring (SSM)	Monitor the actuator speed not to exceed the maximum speed limit.
	Safe Speed Range (SSR)	Monitor and maintaining the actuator to move with a defined speed range only.
Safe Positio	Safely-limited Position (SLP)	Preventing exceeding position limit value. Safe stop is triggered by the violation.
	Safe CAM (SCA)	Monitor and maintaining the actuator to move in a defined position range only.
Safe Torque	Safely limited Torque (SLT)	Preventing the actuator to exceed the torque limit.
Safe Guarding	Danger Field Entry (DFE)	Monitor the human position with safety sensor while entering a defined danger area.
	Manual Reser (MAR)	Manual restart button to ensuring that the process could be continued after inexistence of the human in the danger area.
	CFEX	Monitoring the human entry on the X-th cooperation field
	Minimal Distance (MID)	Monitoring the minimal relative distance between human centre point with robot TCP.
	Sequence Wrong (SWR)	Monitoring the sequence of the working task between human and
	Gesture Control Start (GCS)	Monitoring if the gesture control has been activated
	Gesture Control End (GCE)	Monitoring if the gesture control has been deactivated
	Handing Over Start (HOS)	Monitoring if the handing over has been activated
	Handing Over End (HOE)	Monitoring if the handing over has been deactivated
	Path Planning Start (PPS)	Monitoring if the path planning has been activated
	Path Planning End (PPE)	Monitoring if the path planning has been deactivated
	Flange Control Start (FCS)	Monitoring if the flange control has been activated
	Flange Control End (FCE)	Monitoring if the flange control has been deactivated

Table 1. required safety functions

Appendix II – Safety functions of clustered collaborative operation modes in relation to Interaction levels

Interaction Level	Clustered Collaborative Operation Modes	Collaborative Operation Modes					Safety Functions											
		SRMS	SSM	HG		PFL	Safe Standstill					Safe Motion				Safe Position		Safe Torque
				GestureCtrl	FlangeCtrl		SS1	SBC	STO	SS2	SOS	SLS	SSM	SRS	SDI	SLP	SCA	SLT
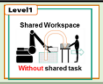	Cluster 1	X	-	-	-	-	X	X	X	-	-	-	-	-	-	-	-	-
	Cluster 2	X	X	-	-	-	X	X	X	-	-	X	X	X	X	-	-	-
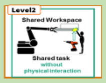	Cluster 1	X	-	-	-	-	X	X	X	X	X	-	-	-	-	-	-	-
	Cluster 2	X	X	-	-	-	X	X	X	X	X	X	X	X	X	-	-	-
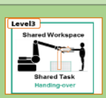	Cluster 1 (GestureControl)	X	X	X	-	-	X	X	X	X	X	X	X	X	X	X	X	-
	Cluster 2 (HandingOver)	-	X	-	-	X	X	X	X	-	-	X	X	X	X	-	-	X
	Cluster 3 (PathPlanning)	X	X	-	-	-	X	X	X	X	X	X	X	X	X	X	X	-
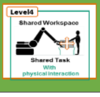	Cluster 1	X	-	-	X	-	-	-		X	X	X	-	-	-	-	-	-
	Cluster 2	-	-	-	X	X	X	X	X	-	-	X	-	-	-	-	-	X
	Cluster 3	X	X	-	X	-	-	-	-	X	X	X	X	X	X	-	-	-
	Cluster 4	-	X	-	X	X	X	X	X	-	-	X	X	X	X	-	-	X

Table 2. Fundamental safety functions

Interaction Level	Clustered Collaborative Operation Modes	Safety Functions														Robot Control					
		Safeguarding														Fixed Path	Speed Ctrl	Gesture Ctrl	Handing Over Ctrl	Path Planning ctrl	Flange Ctrl
		DFE	MAR	CFE1	CFEx	MID	SWR	GCS	GCE	HOS	HOE	PPS	PPE	PCS	FCE						
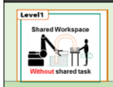	Cluster 1	X	X	-	-	-	-	-	-	-	-	-	-	-	-	X	-	-	-	-	-
	Cluster 2	X	X	X	X	X	-	-	-	-	-	-	-	-	-	X	X	-	-	-	-
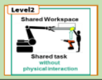	Cluster 1	X	X	-	-	-	X	-	-	-	-	-	-	-	-	X	-	-	-	-	-
	Cluster 2	X	X	X	X	X	X	-	-	-	-	-	-	-	-	X	X	-	-	-	-
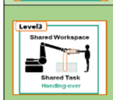	Cluster 1 (GestureControl)	X	X	X	X	X	X	X	X	-	-	-	-	-	-	X	X	X	-	-	-
	Cluster 2 (HandingOver)	-	X	X	X	X	-	-	-	X	X	-	-	-	-	X	X	-	X	-	-
	Cluster 3 (PathPlanning)	X	X	X	X	X	X	-	-	-	-	X	X	-	-	X	X	-		X	-
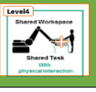	Cluster 1	X	-	-	-	-	-	-	-	-	-	-	-	X	X	X	-	-	-	-	X
	Cluster 2	-	X	-	-	-	-	-	-	-	-	-	-	X	X	X	-	-	-	-	X
	Cluster 3	X	-	X	X	X	-	-	-	-	-	-	-	X	X	X	X	-	-	-	X
	Cluster 4	-	X	X	X	X	-	-	-	-	-	-	-	X	X	X	X	-	-	-	X

Table 3. Safeguarding safety functions and possible types of robot control

9 Appendix III – Safety-Related Finite-State Machine

Level1: Shared Task without shared workspace

Cluster1 – SRMS Standalone

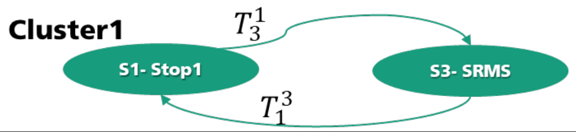

$T_1^3 \rightarrow (DFE \wedge SS1 \wedge SBC \wedge STO)$

$T_3^1 \rightarrow (\overline{DFE} \wedge MAR)$

Cluster 2 – SRMS + SSM

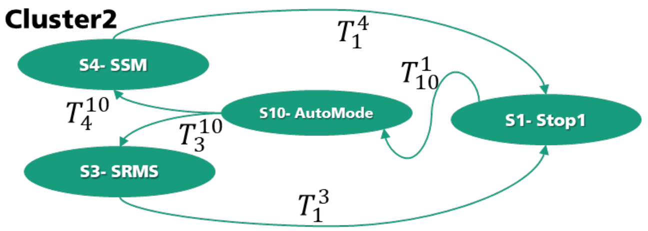

$T_1^3 \rightarrow (DFE) \wedge SS1 \wedge SBC \wedge STO$

$T_1^4 \rightarrow (DFE) \vee (\overline{SLS} \vee \overline{SSM} \vee \overline{SSR} \vee \overline{SDI}) \wedge SS1 \wedge SBC \wedge STO$

$T_{10}^1 \rightarrow (\overline{DFE} \wedge OPR)$

$T_3^{10} \rightarrow (\overline{DFE} \wedge MAR)$

$T_4^{10} \rightarrow \rightarrow (CFE1 \ldots CFEX \wedge SLS \wedge SSM \wedge SSR \wedge SDI)$

Level2 – Shared workspace with shared task (without physical interaction)

Cluster 1: SRMS Standalone

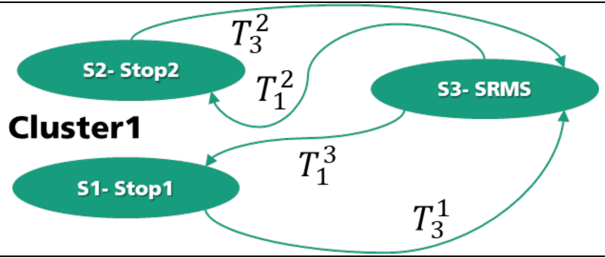

$T_1^3 \rightarrow (DFE \wedge SWR \wedge SS1 \wedge SBC \wedge STO)$

$T_1^2 \rightarrow (DFE \wedge \overline{SWR} \wedge SS2 \wedge SOS)$

$T_3^1 \rightarrow (\overline{DFE} \wedge MAR)$

$T_3^2 \rightarrow (\overline{DFE} \wedge \overline{MAR})$

Cluster 2: SRMS + SSM

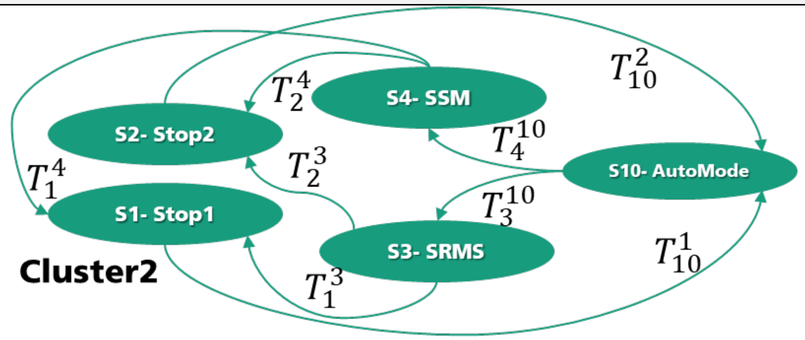

$T_1^3 \rightarrow (DFE \wedge SWR) \wedge SS1 \wedge SBC \wedge STO$

$T_1^4 \rightarrow (DFE \wedge SWR) \vee (\overline{SLS} \vee \overline{SSM} \vee \overline{SSR} \vee \overline{SDI}) \wedge SS1 \wedge SBC \wedge STO$

$T_2^4 = T_2^3 \rightarrow (DFE \wedge \overline{SWR} \wedge SS2 \wedge SOS)$

$T_{10}^1 \rightarrow (\overline{DFE} \wedge OPR)$

$T_{10}^2 \rightarrow (\overline{DFE} \wedge \overline{OPR})$

$T_3^{10} \rightarrow (\overline{DFE} \wedge MAR)$

$T_4^{10} \rightarrow (CFE1 \ldots CFEX \wedge SLS \wedge SSM \wedge SSR \wedge SDI)$

Level3 – Shared workspace with shared task (active robot control)

Cluster 1: SSM + HG@GestureCntrl + SRMS

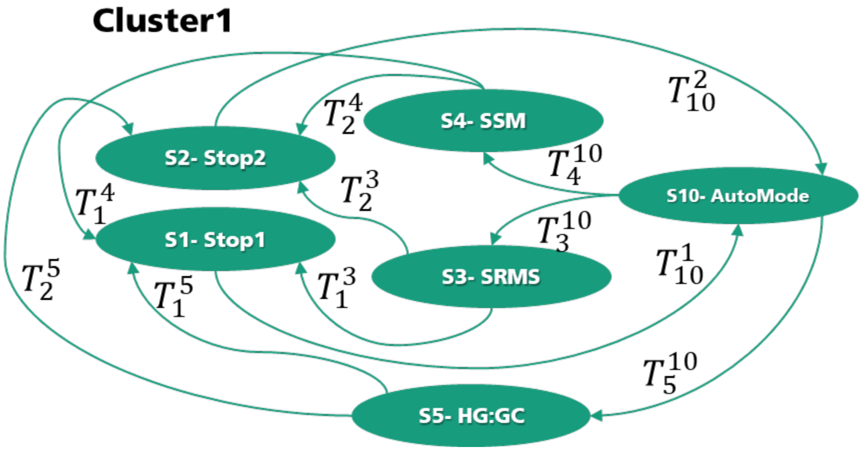

$T_1^3 \rightarrow (DFE \wedge SWR) \wedge SS1 \wedge SBC \wedge STO$

$T_1^4 \rightarrow (DFE \wedge SWR) \vee (\overline{SLS} \vee \overline{SSM} \vee \overline{SSR} \vee \overline{SDI}) \wedge SS1 \wedge SBC \wedge STO$

$T_1^5 \rightarrow (DFE \wedge SWR) \vee (\overline{SLS} \vee \overline{SSM} \vee \overline{SSR} \vee \overline{SDI} \vee \overline{SLP} \vee \overline{SCA}) \wedge SS1 \wedge SBC \wedge STO$

$T_2^5 = T_2^4 = T_2^3 \rightarrow (DFE \wedge \overline{SWR} \wedge SS2 \wedge SOS)$

$T_{10}^1 \rightarrow (\overline{DFE} \wedge OPR)$

$T_{10}^2 \rightarrow (\overline{DFE} \wedge \overline{OPR}) \vee (GCE)$

$T_3^{10} \rightarrow (\overline{DFE} \wedge MAR)$

$T_4^{10} \rightarrow (CFE1 \ldots CFEX \wedge SLS \wedge SSM \wedge SSR \wedge SDI)$

$T_5^{10} \rightarrow (CFEHG \wedge SLS \wedge SSM \wedge SSR \wedge SDI \wedge GCS \wedge SLP \wedge SCA)$

Cluster 2: SSM + PFL + HandingOver

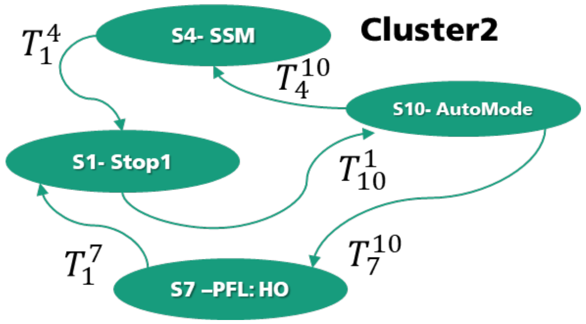

$T_1^4 \rightarrow (\overline{SLS} \vee \overline{SSM} \vee \overline{SSR} \vee \overline{SDI}) \wedge SS1 \wedge SBC \wedge STO$

$T_1^7 \rightarrow (\overline{SLT} \vee \overline{SLS} \vee \overline{SSM} \vee \overline{SSR} \vee \overline{SDI}) \wedge SS1 \wedge SBC \wedge STO$

$T_{10}^1 \rightarrow (\overline{SLT} \wedge OPR) \vee (\overline{SLT} \wedge HOE)$

$T_4^{10} \rightarrow (CFE1 \ldots CFEX \wedge SLS \wedge SSM \wedge SSR \wedge SDI)$

$T_7^{10} \rightarrow (CFEHO \wedge SLS \wedge SSM \wedge SSR \wedge SDI \wedge HOS \wedge SLT)$

Cluster 3: SSM + SRMS + PathPlan

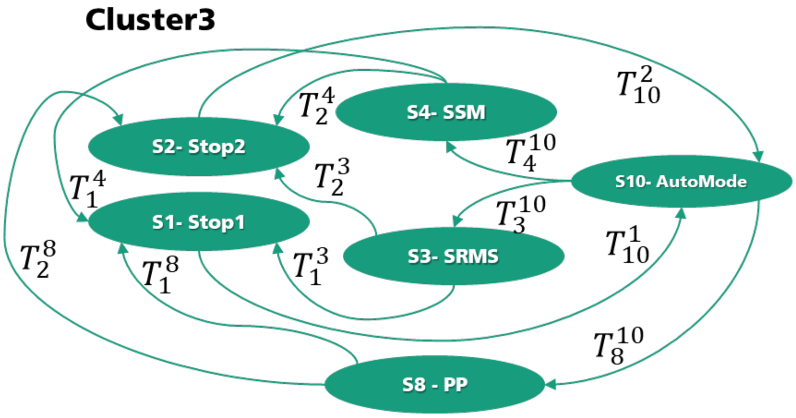

$T_1^3 \rightarrow (DFE \wedge SWR) \wedge SS1 \wedge SBC \wedge STO$

$T_1^4 \rightarrow (DFE \wedge SWR) \vee (\overline{SLS} \vee \overline{SSM} \vee \overline{SSR} \vee \overline{SDI}) \wedge SS1 \wedge SBC \wedge STO$

$T_1^8 \rightarrow (DFE \wedge SWR) \vee (\overline{SLS} \vee \overline{SSM} \vee \overline{SSR} \vee \overline{SDI} \vee \overline{SLP} \vee \overline{SCA}) \wedge SS1 \wedge SBC \wedge STO$

$T_2^8 = T_2^4 = T_2^3 \rightarrow (DFE \wedge \overline{SWR} \wedge SS2 \wedge SOS)$

$T_{10}^1 \rightarrow (\overline{DFE} \wedge OPR)$

$T_{10}^2 \rightarrow (\overline{DFE} \wedge \overline{OPR}) \vee (PPE)$

$T_3^{10} \rightarrow (\overline{DFE} \wedge MAR)$

$T_4^{10} \rightarrow (CFE1 \ldots CFEX \wedge SLS \wedge SSM \wedge SSR \wedge SDI)$

$T_8^{10} \rightarrow (CFEPP \wedge SLS \wedge SSM \wedge SSR \wedge SDI \wedge PPS \wedge SLP \wedge SCA)$

Level4 – Shared workspace with shared task with physical interaction

Cluster 1: SRMS + HG@FlangeCntrl

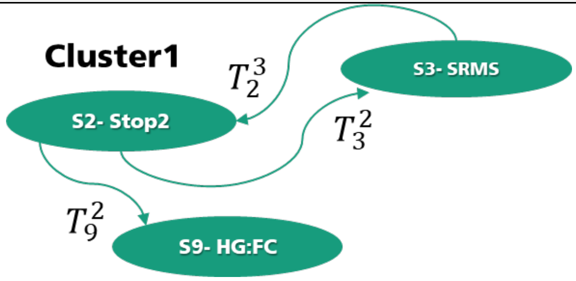

$T_9^2 \rightarrow (FCS \wedge SLS)$

$T_2^3 \rightarrow (DFE \wedge SS2 \wedge SOS)$

$T_3^2 \rightarrow (\overline{DFE} \vee FCE)$

Cluster 2: PFL + HG@FlangeCntrl

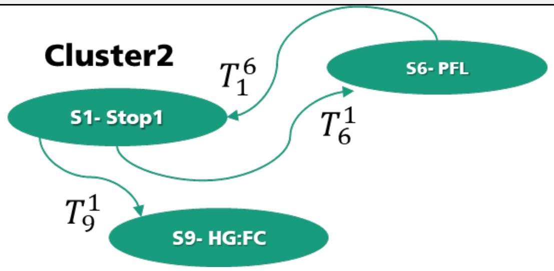

$T_9^1 \rightarrow (FCS \wedge SLS)$

$T_1^6 \rightarrow (SLT \wedge SS1 \wedge SBC \wedge STO)$

$T_6^1 \rightarrow (\overline{SLT} \vee MAR) \vee FCE$

Cluster 3: SSM + SRMS + HG@FlangeCntrl

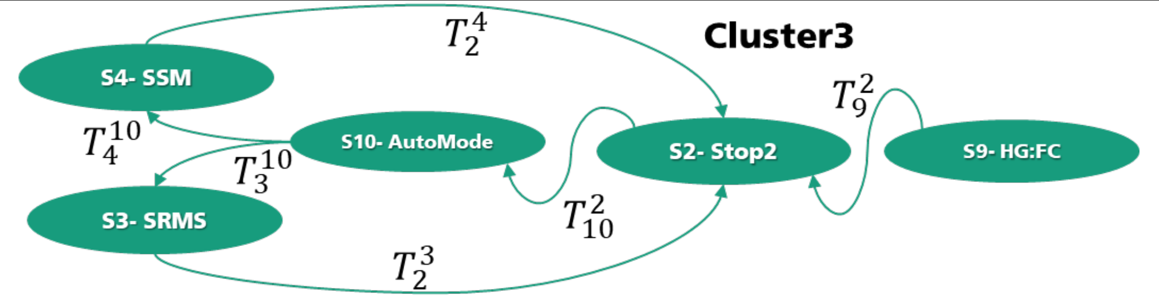

$T_2^4 = T_2^3 \rightarrow (DFE) \wedge SS2 \wedge SOS$

$T_{10}^2 \rightarrow (\overline{DFE} \wedge OPR) \vee FCE$

$T_3^{10} \rightarrow (\overline{DFE} \wedge MAR)$

$T_4^{10} \rightarrow \rightarrow (CFE1 \ldots CFEX \wedge SLS \wedge SSM \wedge SSR \wedge SDI)$

$T_9^2 \rightarrow (FCS \wedge SLS)$

Cluster 4: SSM + PFL + HG@FlangeCntrl

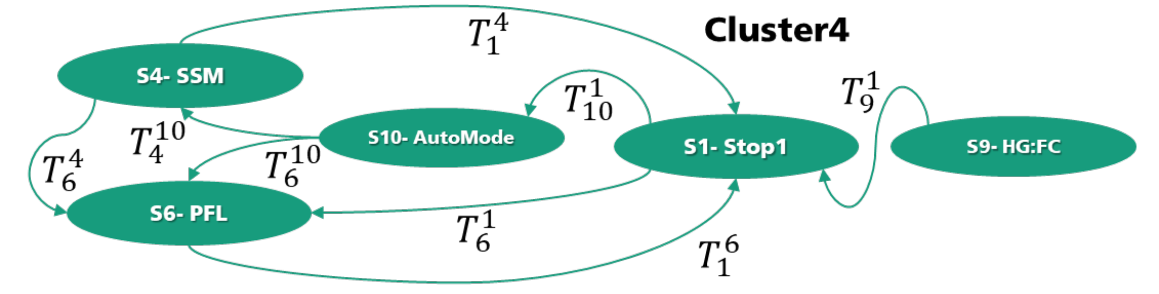

$T_1^4 = T_1^6 \rightarrow (SLT) \wedge SS1 \wedge SBC \wedge STO$

$T_6^4 \rightarrow (DFE)$

$T_6^1 \rightarrow (MAR)$

$T_{10}^1 \rightarrow (\overline{SLT} \wedge OPR) \vee$ FCE

$T_6^{10} \rightarrow (MAR)$

$T_4^{10} \rightarrow \rightarrow (CFE1 \ldots CFEX \wedge SLS \wedge SSM \wedge SSR \wedge SDI)$

$T_9^1 \rightarrow (FCS \wedge SLS)$

Table 4. Safety-Related Finite-State Machine for all interaction levels
